# Epigenetic regulation in prokaryotes: transcriptional and phenotypic outcomes of DNA methyltransferase activity

**DOI:** 10.1093/femsre/fuag014

**Published:** 2026-03-24

**Authors:** Sara Sharaf, Martina Cappelletti, Marco R Oggioni, Karolin Hijazi

**Affiliations:** School of Medicine, Medical Sciences and Nutrition, University of Aberdeen, Aberdeen AB25 2ZD, United Kingdom; Department of Pharmacy and Biotechnology, University of Bologna, 40126 Bologna, Italy; Department of Pharmacy and Biotechnology, University of Bologna, 40126 Bologna, Italy; IRCCS Azienda Ospedaliero-Universitaria di Bologna, 40138 Bologna, Italy; School of Medicine, Medical Sciences and Nutrition, University of Aberdeen, Aberdeen AB25 2ZD, United Kingdom

**Keywords:** epigenetics, restriction–modification systems, DNA methyltransferases, DNA methylation, prokaryotes, bacteria

## Abstract

DNA methyltransferases (DNA MTases) are central epigenetic regulators in bacteria and archaea, with functions extending far beyond classical restriction–modification defence. Diverse MTase classes exist, including canonical and phase-variable restriction–modification systems, orphan MTases, and enzymes with currently undefined roles. MTase activity is associated with regulatory outcomes through both direct and indirect mechanisms. Methylation of promoters or regulatory regions can influence transcription, while broader methylome remodelling may affect genome-wide gene expression. These processes generate distinct epigenetic states associated with phenotypic variation. MTase-mediated regulation has been implicated in virulence, colonization, immune evasion, biofilm formation, motility, stress tolerance, metabolism, and antibiotic susceptibility. In archaea, MTase systems contribute to genome integrity and ecological specialization, highlighting shared epigenetic principles across domains of life. A major challenge is to move beyond descriptive methylome surveys and correlative analyses toward experimentally validated links between methylation and phenotype. This review synthesizes current understanding of prokaryotic DNA methylation, with primary emphasis on experimentally validated phenotypic outcomes. We also incorporate insights from omics-based studies where these provide context or generate testable hypotheses, while noting when evidence is based on inference rather than direct experimental validation, and include underrepresented archaeal systems.

## Teaser

This review shows how DNA methylation acts as a regulatory layer in bacteria and archaea, shaping diverse biological traits and behaviours.

## Introduction

Epigenetics encompasses reversible changes in gene expression that occur without alteration of the DNA sequence itself. DNA methylation has long been recognized as a major epigenetic mechanism, but its roles and evolutionary significance differ markedly between eukaryotes and prokaryotes (Willbanks et al. 2016). In eukaryotes, methylation occurs predominantly at cytosine residues within CpG dinucleotides and functions as a genome-wide regulatory system that contributes to transcriptional silencing, imprinting, and chromatin organization (Bird 2002, Law and Jacobsen 2010, Moore et al. 2013). These modifications act in concert with histone marks and chromatin remodeling complexes to modulate stable yet reversible gene expression states essential for development and differentiation. Eukaryotic methylation patterns are often inherited through cell division and are tightly regulated by DNA methyltransferases such as DNMT1, DNMT3A, and DNMT3B in mammals which highlights its central role in maintaining cellular integrity and function (Meng et al. 2024).

By contrast, DNA methylation in prokaryotes was historically viewed mainly through the framework of restriction–modification (R–M) systems, where methylation acts as a self-recognition signal that blocks restriction enzyme cleavage of the host genome. This defense-oriented perspective long obscured the broader regulatory potential of bacterial DNA methyltransferases (MTases) (Rodic et al. 2017, Shaw et al. 2023). The identification of orphan MTases, enzymes lacking an associated restriction endonuclease marked a major conceptual shift. Classic examples such as Dam and Dcm in *Escherichia coli* and CcrM in *Caulobacter crescentus* revealed that DNA methylation can directly modulate bacterial physiology (Wenzel and Guschlbauer 1993, Løbner-Olesen et al. 2005, Horton et al. 2019).

Recent advances in single-molecule real-time (SMRT) sequencing have demonstrated that MTases are widespread across bacterial and archaeal lineages (Tourancheau et al. 2021, Chen et al. 2022). Prokaryotic MTases typically generate N6-methyladenine (6mA), N4-methylcytosine (4mC), or 5-methylcytosine (5mC), thereby modifying specific motifs and impacting downstream phenotypic characteristics through changes in gene expression patterns (Sánchez-Romero et al. 2015). Beyond orphan systems, phase-variable MTases are enzymes whose activity or DNA-recognition specificity can change through reversible genetic alterations usually associated with type I and type III R–M loci form “phasevarions” that mediate coordinated and reversible regulation of multiple genes. However, regulatory activity is not limited to phase-variable systems; several non-phase-variable Type I and Type II MTases can modulate gene expression and contribute to variation in virulence and other phenotypic traits (Anton and Roberts 2021). Such systems contribute to the pathogenicity of bacterial species such as *Neisseria, Haemophilus*, and *Helicobacter* (Donahue et al. 2002, Atack et al. 2019, Seib et al. 2020).

Collectively, these insights position DNA methylation as a multilayered regulatory mechanism that links environmental cues to dynamic gene expression programs and extends far beyond its originally recognized role in host defense (Sánchez-Romero et al. 2015, Seib et al. 2020, Seong et al. 2021). It is increasingly clear that DNA methylation can influence gene expression by modulating promoter accessibility, altering transcription factor binding, or affecting DNA topology, ultimately contributing to phenotypic heterogeneity and environmental adaptability (Passeri et al. 2024, Chen et al. 2025).

Although several reviews have described the diversity of prokaryotic DNA methyltransferases and their biochemical properties (De Ste Croix et al. 2017, Phillips et al. 2019, Seong et al. 2021, Anton and Roberts 2021, Payelleville and Brillard 2021, Gao et al. 2023, Chen et al. 2025), and others have examined bacterial phenotypes primarily through transcriptomic profiling (Ma et al. 2025b), a unifying perspective on the functionally validated consequences of DNA methylation across bacterial and archaeal systems remains lacking. Recent advances in genome-wide methylome profiling, coupled with expanding experimental evidence, now make it possible to directly link methylation states to cellular physiology, genome stability, and ecological adaptation. Here, we synthesize these developments to provide a function-oriented framework for understanding how DNA methylation shapes prokaryotic biology. We place primary emphasis on studies in which phenotypic outcomes have been experimentally validated, while also incorporating selected insights from metagenomic and other omics-based studies where these provide useful context or generate testable hypotheses. Such studies are clearly distinguished from experimentally validated evidence throughout.

By integrating evidence across diverse systems, we highlight how DNA methylation influences ecological adaptability, host-microbe interactions, and phenotypic heterogeneity, often through underlying molecular regulatory processes. In doing so, this review moves beyond enzyme classification and predominantly correlative omics-based approaches to better delineate causal relationships and provide a cohesive view of how DNA methylation contributes to adaptive evolution in prokaryotes.

## Diversity and classification of prokaryotic MTases

### Canonical and phase-variable restriction–modification systems

R–M systems are ubiquitous across bacterial and archaeal lineages and represent one of the earliest described mechanisms of prokaryotic DNA methylation. Traditionally regarded as innate immune systems, R–M loci protect bacterial hosts against invasion by foreign genetic material, such as bacteriophages and plasmids, by discriminating between self and non-self DNA (Vasu and Nagarajan 2013). These systems are composed of a restriction endonuclease (R) that cleaves foreign unmethylated DNA and a corresponding methyltransferase (M) that methylates host DNA at specific recognition motifs, preventing autodegradation. R–M systems have been also reported to be spread through horizontal gene transfer, possibly contributing to bacterial genome plasticity and adaptation under environmental pressures (Tisza et al. 2023, Gopalan-Nair et al. 2024).

Four canonical types of R–M systems (Types I-IV) have been characterized, each distinguished by their subunit composition, sequence recognition motifs, cleavage positions, and cofactor requirements (Raleigh and Brooks 1998). Type I systems, encoded by the *hsdR, hsdM*, and *hsdS* genes, form multi-subunit protein complexes comprising restriction (R), methylation (M), and specificity (S) subunits. The specificity subunits denote the target motif which is usually bipartite in nature due to the specificity subunit arrangement within the operon. They utilize ATP to cleave DNA at variable distances from their recognition sites, linking restriction activity with energy-dependent DNA translocation (Loenen et al. 2014). Type II systems, in contrast, consist of independent restriction and methylation enzymes that recognize short, palindromic sequences and cleave within or near these targets. Their high specificity and predictable cleavage patterns have made them invaluable tools in molecular biology and biotechnology, EcoRI is a well-substantiated example that Type II enzymes serve as foundational tools for molecular cloning and DNA manipulation. (Chang and Cohen 1977, Pingoud et al. 2014, Wang et al. 2021). Type III systems are composed of two genes, *mod* (modification) and *res* (restriction), which encode subunits that operate as a hetero-oligomeric complex. These enzymes recognize asymmetric sequences, require ATP for cleavage, and translocate DNA in a unidirectional manner prior to restriction (Rao et al. 2014). Type IV systems differ from the rest, as they contain a restriction gene but lack a methyltransferase, they target and cleave modified DNA, thereby countering host-like methylation in invading phage genomes (Loenen et al. 2014).

While canonical R–M systems were long considered purely defensive, accumulating evidence suggests that some MTases within these systems, particularly phase-variable enzymes, can exert epigenetic regulatory functions. Phase-variable methylation arises either through ON/OFF expression of an MTase (common in Type III *mod* genes though limited representation exists in Type II systems) or through changes in target specificity (*hsdS* recombination in Type I systems). These molecular rearrangements alter the DNA motifs they recognize, producing distinct methylation signatures that, in turn, generate different gene expression profiles across host subpopulations in response to environmental cues (Srikhanta et al. 2009, Manso et al. 2014, Anjum et al. 2016). This is demonstrated in Type III Mod phasevarions of *Neisseria, Haemophilus*, and *Moraxella* species, where reversible ON/OFF expression of the *mod* gene results in global shifts in methylation patterns and downstream transcriptional regulation of multiple genes (Fox et al. 2007, Kwiatek et al. 2015, Blakeway et al. 2019). Similarly, Type I phasevarions, such as those found in *Streptococcus pneumoniae* and *Neisseria gonorrhoeae*, can modulate gene expression networks by stochastic changes in the specificity subunit (*hsdS*) due to recombination between target recognition domains (Manso et al. 2014, Adamczyk-Poplawska et al. 2022).

Therefore, canonical MTases within R–M systems, especially phase-variable ones, serve not only as components of genome defense but also as modulators of transcription and phenotypic plasticity. With this dual regulatory capacity established for canonical R–M systems (De Vries et al. 2002, Bower et al. 2018, Shaw et al. 2023), this foundation sets the stage for understanding how orphan and other non-canonical and undefined methyltransferases contribute to epigenesis and broader methylome-driven regulation in prokaryotes.

### Orphan methyltransferases and regulatory specialization

Orphan DNA MTases constitute a distinct class of bacterial methyltransferases that function independently of restriction endonucleases, representing a shift from defensive methylation toward primarily regulatory epigenetic roles. Although some comparative analyses suggest that certain orphan MTases may have originated through loss or inactivation of cognate restriction endonucleases, the evolutionary history of orphan MTases remains unresolved and likely varies across lineages (Seshasayee et al. 2012).

Well-characterized systems adopted across diverse prokaryotic taxa such as Dam in *E. coli* which targets GATC sites but can interact with non-canonical sites influencing key processes such as replication initiation, mismatch repair, nucleoid organization, and the transcription of virulence and stress-response genes (Wyrzykowski et al. 2003, Horton et al. 2015). Although less well characterized, Dcm in *E. coli* targets CCWGG sites and contributes to mutation avoidance and can modulate gene expression under specific stress conditions (Guss et al. 2012, Militello et al. 2012). In *C. crescentus*, the temporally controlled CcrM MTase targeting GANTC sites exemplifies a cell cycle-regulated epigenetic system that coordinates replication with the activation of differentiation-associated genes (Reisenauer et al. 1999, Horton et al. 2019).

The essential nature of these enzymes is further highlighted by the association with cell viability in *Yersinia pseudotuberculosis* and *Vibrio cholerae* (Dam-like MTase), and in *Agrobacterium tumefaciens* and *Caulobacter crescentus* (CcrM-like MTase) (Julio et al. 2001, Kahng and Shapiro 2001, Gonzalez et al. 2014). Loss or dysregulation of these MTases results in severe replication defects, aberrant cell-cycle progression, compromized DNA repair, and defects in maintaining cellular homeostasis.

### Undefined and novel methyltransferases revealed by methylome profiling

**Figure 1:**
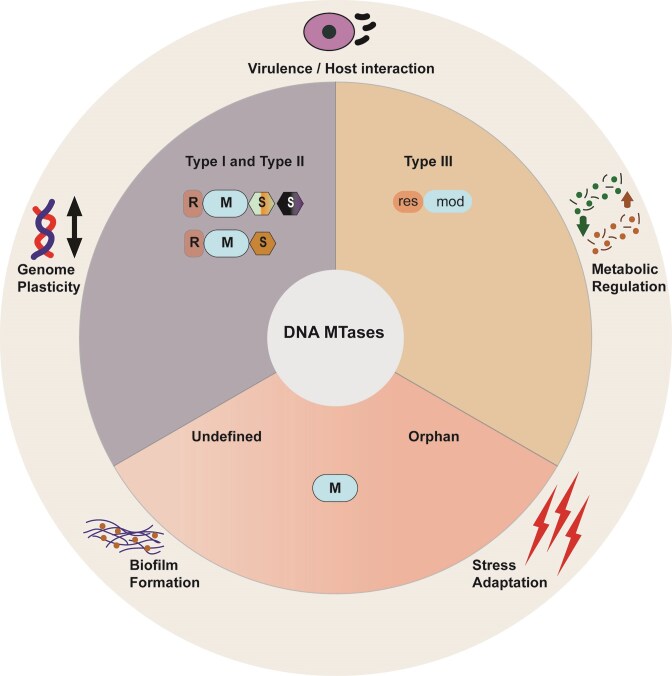
Functional landscape of prokaryotic DNA methyltransferase systems. Schematic representation of major classes of DNA methyltransferases (MTases) in prokaryotes and the breadth of functional consequences associated with their activity. The central diagram illustrates principal MTase categories, including canonical R–M systems (Type I/II and Type III), orphan MTases that lack cognate restriction endonucleases but possess established regulatory roles, and undefined MTases identified through genome-wide methylome profiling. Undefined MTases are shown with lighter shading to reflect that their biological functions remain incompletely characterized despite detectable methylation activity. Abstract symbols denote methyltransferase (M), restriction (R), and specificity (S) subunits for Type I and II systems, and restriction (*res*) and modification (*mod*) in Type III systems. These are intended to convey functional composition rather than accurate structural organization. Phase-variable behaviour is not depicted as a discrete class but is implicit across multiple MTase categories, reflecting reversible changes in methyltransferase expression or specificity that generate alternative methylation states. The outer ring summarizes major functional domains influenced by DNA methylation, including virulence and host interaction, metabolic regulation, stress adaptation, biofilm formation, and genome plasticity. The figure emphasizes that individual methylation systems frequently contribute to multiple phenotypic outcomes and that similar biological functions can arise from distinct methylation architectures. Together, this framework highlights DNA methylation as a modular and integrative regulatory system extending beyond classical restriction-based genome defense to shape diverse aspects of prokaryotic physiology and adaptation.

Single-molecule real-time (SMRT) and nanopore sequencing have revolutionized the exploration of prokaryotic methylomes, uncovering vast numbers of MTases with no clear homology to characterized families (Beaulaurier et al. 2015, Chen et al. 2022). With continued development of supporting bioinformatic tools that capitalize on these SMRT technologies, genome-wide mapping of methylation motifs and their assignment to cognate MTases at higher resolution and depth has been made possible (Flusberg et al. 2010, Clark et al. 2012, Blow et al. 2016). These findings indicate that our current classification schemes largely derived from bacterial R–M systems likely capture only a subset of existing methylation diversity. Many of these “undefined” or “unassigned” MTases appear particularly abundant in archaeal genomes, where they may exhibit unique combinations of catalytic and accessory domains (Hiraoka et al. 2022). In particular, archaeal species such as *Sulfolobus solfataricus* and *Methanococcus jannaschii* display unusual modification Msignatures, including noncanonical sequence motifs and novel catalytic domain architectures (Huang et al. 2002, Menezes et al. 2011, Wang et al. 2014). The functional roles of these enzymes remain poorly understood, though their diversity suggests adaptation to niche-specific pressures such as high-temperature survival, viral predation, or horizontal gene exchange (Fullmer et al. 2019). Comparative methylomics across bacterial and archaeal taxa suggests that the repertoire of MTases in a given organism might reflect ecological specialization, pointing toward the acquisition and utilization of these systems as regulatory systems and/or evolutionary drivers (Makarova et al. 2013). In line with this view, several prokaryotic MTase genes have been found to be co-localized with transposases and integrases that mediate genomic plasticity and possible adaptation to extreme or fluctuating environments (Makarova et al. 2013). Although experimental validation remains limited, these observations expand the conceptual boundaries of bacterial and archaeal epigenetics, positioning undefined MTases as a frontier for discovery in understanding how methylation contributes to cellular regulation and evolution (Harris and Goldman 2020, Hiraoka et al. 2022). The diversity of prokaryotic DNA methyltransferase systems and the breadth of functional outcomes they influence are summarized in Fig. 1.

## Mechanistic links between methylation and gene expression

### Direct mechanisms of methylation-driven gene expression

**Figure 2:**
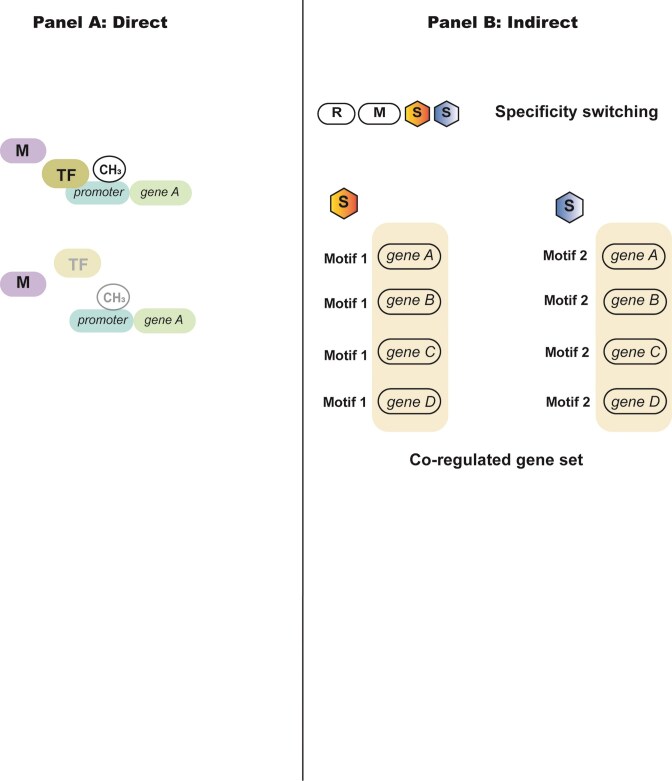
Direct and indirect mechanisms by which DNA methylation modulates gene regulation in prokaryotes. Schematic overview of two principal mechanistic modes through which DNA methylation influences gene expression in prokaryotes. Panel A (Direct): Methylation at defined loci directly modulates DNA–protein interactions at individual promoters. Methylation of promoter-associated motifs can inhibit or permit transcription factor (TF) binding, thereby altering transcriptional output at specific genes in a reversible and heritable manner. Panel B (Indirect): Changes in methyltransferase activity, expression, or target specificity generate distinct genome-wide methylation landscapes. Specificity switching or ON/OFF expression of methyltransferases reconfigures methylation across multiple loci, producing coordinated changes in expression among sets of genes without direct promoter-specific targeting. These mechanisms are not mutually exclusive and can coexist within a single organism, collectively contributing to differential phenotypes and complex regulatory outcomes.

Direct methylation-dependent regulatory mechanisms frequently but not exclusively operate at specific gene promoters, where methylation can influence transcriptional factor binding to DNA. These locus-specific effects arise from direct methylation-dependent modulation of DNA-protein interactions at promoters, as schematically illustrated in Fig. 2A.

Although DNA methylation systems in prokaryotes have been extensively catalogued, the mechanistic basis by which methylation influences gene regulation remains unresolved in many taxa. In numerous instances, it is difficult to attribute transcriptional outcomes to a single, direct methylation-dependent mechanism, and the available evidence instead points toward hybrid or predominantly indirect modes of regulation. A growing body of work supports the view that DNA methyltransferases (MTases) can modulate transcriptional programs without acting as classical site-specific regulators. In many systems, however, transcriptional changes following MTase perturbation are difficult to attribute to discrete methylation-sensitive binding events. Instead, phenotypes emerging from MTase knockout or overexpression often reflect broader physiological rewiring, altered replication timing, stress state, or nucleoid organization that secondarily reshapes gene expression (Løbner-Olesen et al. 2003). However, there are examples of clear site-specific methylation-driven modulation.

The *agn43* and *pap* genes are among the best-characterized examples of direct site-specific phase variation in *E. coli*. The process of phase variation is integral to microbial adaptation; it involves a heritable and reversible high frequency genetic event that leads to sequence shuffling and leads to in-population phenotypic heterogeneity. The gene *agn43* encodes a self-recognizing, a fimbrial adhesin that promotes cell aggregation, biofilm formation, and oxidative stress resistance, contributing to survival within host neutrophils (Haagmans and van der Woude 2000). Its expression is governed by a Dam-dependent phase-variable switch, generating phase-ON and phase-OFF populations. Activation depends on DNA adenine methylation event by Dam, whereas repression requires the transcriptional regulator OxyR. OxyR binding protects specific GATC sites from Dam methylation, thereby preventing transcription (Waldron et al. 2002, Cabrer-Panes et al. 2020). The mutually exclusive binding of Dam and OxyR to overlapping target sequences establishes a competitive regulatory circuit in which methylation status determines promoter accessibility and transcriptional state. In *Salmonella enterica*, a comparable methylation-based switch regulates the *gtr* (glycosyltransferase) operon responsible for O-antigen modification (Broadbent et al. 2010). Again, OxyR mediates the transition between ON and OFF states by binding to sites whose occupancy is dictated by the methylation status of adjacent GATC motifs. This interplay between OxyR binding and Dam-dependent methylation enables reversible control of virulence-associated surface modifications (Broadbent et al. 2010).

A similar Dam-mediated ON/OFF regulatory mechanism governs the pyelonephritis-associated pili (*pap*) operon, which controls fimbrial expression and adhesion potential in *E. coli*. Here, transcription is controlled by the promoter binding of global regulator Leucine-responsive regulatory protein Lrp and local regulatory protein PapI and is governed by the methylation state of two GATC sites namely GATC1028 and GATC1130 which represents a proximal and distal site within the *pap* regulatory region (Kaltenbach et al. 1995, Hale et al. 1998, Braaten et al. 1994)). When the proximal site is fully methylated, the Lrp/PapI complex binds cooperatively and the *pap* genes are transcribed “phase-ON”, when the distal site is fully methylated by Dam, only Lrp is bound at a different site preventing transcription and resulting in a “phase-OFF” state. Together, these interactions maintain a stable methylation pattern and a heritable transcriptional state.

Other DNA methyltransferases that are not orthologs of Dam can also be involved in gene expression regulation like DnmA in *Bacillus subtilis* (Nye et al. 2020) and CcrM in members of Alphaproteobacteria class. DnmA catalyzes N^6^-adenine methylation (6mA) within a subset of promoter sequences containing a conserved consensus motif. DnmA-driven methylation influences the expression of genes involved in chromosomal structure and maintenance, suggesting a role in coordinating replication and transcription that is still not fully elucidated (Nye et al. 2020). CcrM modulates the activity of promoters involved in the cell-cycle, in various alphaproteobacterial species including *C. crescentus, Agrobacterium tumefaciens*, and *Sinorhizobium meliloti (formerly Rhizobium meliloti)* (Wright et al. 1997). CcrM operates through a controlled temporal mechanism, expressed only once per cell cycle, before cell division and is rapidly degraded thereafter. This regulation establishes cyclic methylation patterns; wherein newly replicated DNA is hemimethylated and becomes fully methylated only upon the reappearance of CcrM in the subsequent cycle (Stephens et al. 1996). Within this framework, the global transcriptional regulator GcrA activates cell-cycle-regulated promoters, while CcrM-dependent methylation stabilizes their expression state across the replication cycle (Kahng and Shapiro 2001, Horton et al. 2019). Unlike Dam-based switches that rely primarily on transcription factor competition, CcrM-mediated regulation integrates promoter methylation with replication timing, directly influencing transcription initiation. As a result, CcrM is essential for viability in these bacteria and controls genes involved in DNA replication (*gyrA*), chromosome decatenation (*nstA*), and cell division (*ftsZ*) through methylation of GANTC motifs in their promoter regions (Wright et al. 1997, Kahng and Shapiro 2001, Reich et al. 2018). CcrM was initially reported to be essential for viability in *Brucella abortus* and *A. tumefaciens* (Robertson et al. 2000 , Kahng and Shapiro 2001). More recent work in *C. crescentus* has shown that loss of *ccrM* can be bypassed through compensatory mutations in the phosphoenolpyruvate carbohydrate phosphotransferase system (PTS), which provide an alternative route for coordinating *ftsZ* and *mipZ* expression. However, these *ΔccrM* strains are viable only in rich medium and exhibit markedly reduced fitness relative to the wild type (Gonzalez and Collier 2015).

A direct methylation mechanism that does not target promoter regions involves the Dam-mediated SeqA-oriC replication control system in *E. coli*. DNA replication initiates when the initiator protein DnaA binds to specific boxes within the origin of replication (*oriC*), triggering local DNA unwinding and assembly. Immediately after replication, the newly synthesized DNA strand is hemimethylated at GATC motifs, as only the parental strand carries Dam-dependent 6 mA modifications (Nou et al. 1993, Hernday et al. 2002). The SeqA protein specifically recognizes these hemimethylated GATC sites and binds to them, forming nucleoprotein complexes (Han et al. 2004). This SeqA-DNA complex acts to sequester the newly replicated origins, thereby preventing premature reinitiation of replication until Dam methylase restores full methylation of *oriC* (Kang et al. 2005, Nievera et al. 2006). Through this methylation-dependent binding cycle, SeqA ensures that replication occurs only once per cell cycle and maintains proper replication timing. Disruption of this process either by loss of SeqA or aberrant methylation patterns leads to asynchronous initiation events, disordered nucleoid organization, and abnormal DNA supercoiling, whereas SeqA overproduction can impair cell division (Chung YS et al. 2009).

### Indirect mechanisms of methylation-driven gene expression

Indirect mechanisms operate primarily through genome-wide remodelling of methylation patterns rather than through locus-restricted promoter control (Fig. 2B). By redistributing methylation across motifs that occur throughout the chromosome, these systems alter the regulatory landscape and generate coordinated shifts in transcription. Classical examples include the phase-variable R–M systems of Type I and Type III classes, which dynamically reprogram genome-wide methylation states in response to stochastic or environmental signals.

In Type I systems, recombination between variable regions of the *hsdS* gene alters the sequence specificity of the methyltransferase, generating new combinations of DNA-binding motifs. Since Type I methyltransferases do not target promoters directly but instead recognize specific sequence motifs that can occur anywhere in the genome, including promoters, coding regions, intergenic regions, and distal regulatory sites, a change in specificity reshapes the entire pattern of genomic methylation. As a result, the subset of loci that become methylated shifts globally, producing broad changes in transcriptional potential without the need for promoter-specific targeting (Bower et al. 2018). In contrast, Type III *mod* systems generate ON/OFF expression states of the methyltransferase through slipped-strand mispairing or changes in the number of repeated sequences within the *mod* gene. These reversible shifts determine whether the methyltransferase is produced or not, leading to variation in methylation events at its specific recognition sites. The presence or absence of methylation at defined recognition motifs produces alternative methylation profiles, each capable of influencing the DNA-protein interaction and, consequently, the transcriptional profile of the cell (Murray et al. 2021).

Beyond phase-variable systems, indirect methylation effects also emerge through modulation of nucleoid-associated protein (NAP) binding. Widespread methylation can alter local DNA curvature or rigidity, influencing the affinity of structural proteins such as Fis and IHF, which organize chromosomal architecture and regulate processes such as transcription, recombination, and nucleoid compaction (Charlier et al. 1994, Shao et al. 2008). In this way, methylation indirectly governs higher-order chromosomal organization and, by extension, the transcriptional output of large gene clusters.

Dam methylation has been implicated in modulating the PmrA/PmrB and RcsC/RcsD/RcsB two-component systems in *S. enterica* leading to LPS structure modifications (Sarnacki et al. 2013). These regulatory effects likely reflect indirect consequences of altered methylation triggered by environmental cues on global transcriptional networks. Further investigation is required to determine whether this reflects secondary physiological adaptation or a more defined methylation-mediated mechanism.

In *Synechocystis sp*. 6803, a subtype I-D CRISPR-Cas system exhibits 5mC hypermethylation within CRISPR1 repeat-spacer regions and hypomethylation at *cas* genes, primarily at the 5mCGATCG motif targeted by M.Ssp6803I. Although CRISPR interference activity and *cas* gene expression remain unaffected, loss of 5mC methylation reduces conjugation efficiency by ∼50%. This suggests that methylation within CRISPR-cas system contributes to structural or DNA-processing dynamics associated with conjugative DNA transfer rather than directly regulating *cas* transcription (Scholz et al. 2019). The precise mechanism remains unresolved, but these findings point to an indirect, non-transcriptional role for DNA methylation within CRISPR-cas systems.

DNA methylation patterns through direct or indirect modulation underpin the functional and phenotypic outcomes discussed in Section 4.

## Functional and phenotypic consequences of DNA methylation

DNA methylation shapes prokaryotic phenotypes by coupling gene regulation, genome maintenance, and physiological adaptation. These effects can arise through direct, site-specific modulation of transcription or replication, or indirectly through genome-wide methylation driving the regulation of multiple genes across the entire genome. Together, these mechanisms influence host interaction, stress survival, genome evolution, core metabolism, and community-level behaviours. The following subsections illustrate how these methylation-dependent mechanisms manifest across distinct but overlapping functional domains. Where individual studies report effects spanning multiple domains, we discuss them under the primary phenotype emphasized by the authors and cross-reference secondary outcomes elsewhere.

### Virulence and host-pathogen interactions

DNA methylation has emerged as a central regulatory layer shaping bacterial virulence by reshaping gene expression involved in host colonization, persistence, and immune evasion. Across pathogens from human, animal and plant-associated species, MTases remodel the production of adhesins, secreted effectors, and cell-surface structures that collectively determine host-pathogen dynamics. These effects frequently arise through epigenetic phase variation or broader methylome reconfiguration, enabling rapid and reversible phenotypic diversification during infection. Representative examples of methylation-dependent virulence phenotypes across prokaryotes are summarized in Table 1.

**Table 1:** Virulence and host interaction-associated outcomes of DNA methyltransferase (Section 4.1).

MTase category	Species	MTase state/manipulation	Virulence-associated functional outcome	Reference
Phase-variable Type I R–M	*Streptococcus pneumoniae*	Locked *hsdS* alleles	Altered adhesion, persistence, and invasive disease	Manso et al. 2014, Oliver et al. 2017
Phase-variable Type III (ModA13)	*Neisseria gonorrhoeae*	ON/OFF *mod* variants	Adhesion/invasion trade-off during mucosal infection	Srikhanta et al. 2009, Kwiatek et al. 2015
Type I R–M system	*Aeromonas veronii*	Deletion mutant	Loss of motility and reduced host colonization	Ma J et al. 2025
Phase-variable Type III (ModH5)	*Helicobacter pylori*	ON/OFF *mod* variants and deletion mutants	Differential adhesion and motility and colonization	Srikhanta et al. 2011, Gauntlett et al. 2014
Orphan MTase (Dam)	*Actinobacillus actinomycetemcomitans*	Deletion mutant	Enhanced toxin secretion and reduced epithelial invasion	Wu et al. 2006
Orphan MTase (Dam)	*Aeromonas hydrophila*	Overexpression mutant	Attenuation of systemic virulence	Erova et al. 2006
Type I R–M system	*Pseudomonas syringae*	Deletion mutant	Enhanced pathogenicity in plant infection models	Huang et al. 2025
Orphan MTase	*Pseudomonas aeruginosa*	Deletion mutant	Reduced intracellular survival under immune pressure	Han et al. 2022
Phase-variable Type I R–M	*Listeria monocytogenes*	Locked *hsdS* alleles	Distinct niche-specific virulence programs	Zbinden et al. 2020
Phase-variable Type I R–M	*Streptococcus suis*	Locked *hsdS* alleles	Altered virulence and host mortality	Roodsant et al. 2024

A recurring theme across pathogens is methylation-dependent control of adhesion, motility, and tissue tropism, which represent the earliest determinants of successful infection. In *Streptococcus pneumoniae*, phase-variable Type I R–M systems (Spn556II and SpnD39III) generate alternative *hsdS* allelic states that control colony opacity, epithelial adhesion, and nasopharyngeal persistence. Locked allele variants display reproducible differences in adherence to lung and nasopharyngeal epithelial cells, progression to bacteremia, and within-host persistence, demonstrating how epigenetic switching promotes niche adaptation (Manso et al. 2014, Oliver et al. 2017, Phillips et al. 2022, Agnew et al. 2023). Comparable regulation is observed in *Neisseria gonorrhoeae*, where the ModA13 phasevarion governs pilus-associated genes and outer membrane proteins. ModA13-OFF variants show reduced adherence but markedly enhanced invasion of cervical epithelial cells, illustrating how ON/OFF MTase states encode opposing traits relevant to mucosal colonization versus intracellular entry (Srikhanta et al. 2009, Kwiatek et al. 2015). *Campylobacter jejuni* Type II-G R–M system “*cj0031*” exhibited ∼two-fold reduction in adherence and invasion of epithelial cells by *cj0031-OFF* variant and deletion mutant compared to the wildtype *cj0031-ON* (Anjum et al. 2016).

Methylation-dependent modulation of surface-associated traits extends to motility and flagellar assembly. In *Aeromonas veronii*, a canonical Type I R–M system indirectly controls flagellar biosynthesis, such that knockout mutants lose motility and exhibit sharply diminished kidney colonization in murine infection models (Ma et al. 2025a). Notably, the inability to restore flagella through complementation suggests that methylation establishes stable epigenetic states influencing surface-assembly pathways (Ma et al. 2025a). A similar association between methylation and host colonization is evident in *Helicobacter pylori*, where the phase-variable Type III MTase *modH5* exhibited differential colonization of murine models and was found to regulate both outer-membrane adhesins and flagellar genes which are involved in adhesion and motility, respectively (Srikhanta et al. 2011, Gauntlett et all 2014). The *modH5*-OFF strains upregulate the adhesin HopG, whereas the *modH5*-ON variants upregulate the expression of motility-associated genes (Srikhanta et al. 2011). Additionally, screening of clinical isolates indicated the predominance of *mod3* and *mod5* systems (Srikhanta et al. 2011). Phase-variable methylation systems also influence outer membrane protein expression in *Haemophilus influenzae* biogroup *aegyptius*. Two Type III systems, ModA13 and ModA16, produce significant differential expression of outer membrane proteins between ON and OFF states; however, serum-killing assays indicate that only ModA16 contributes to this phenotype, with no significant difference observed between its ON and OFF variants (Tram et al. 2024).

Beyond early host contact, DNA methylation modulates the deployment of virulence factors, including toxins, secretion systems, and effector repertoires. In *Actinobacillus actinomycetemcomitans*, loss of Dam methylation leads to a fourfold increase in leukotoxin secretion, while it reduces epithelial invasion (Wu et al. 2006). In *Aeromonas hydrophila*, Dam overproduction suppresses Type III secretion system-mediated cytotoxicity while inducing hemolysin and protease activity. This leads to an imbalanced virulence state that attenuates systemic infection in mice while preserving intestinal colonization (Erova et al. 2006). Similar methylation-dependent reweighting of virulence programs is observed in plant pathogens. Deletion of the Type I HsdMSR system in *Pseudomonas syringae* dysregulates Type III secretion components and enhances pathogenicity in bean leaf infection models (Huang et al. 2025). While the putative MTases XvDMT2 overexpression in *Xanthomonas euvesicatoria* reduced virulence during tomato plant infection (Park et al. 2021).

In addition to shaping adhesion and virulence factor deployment, DNA methylation strongly influences how pathogens confront host immune defenses and persist within intracellular niches. A central recurring mechanism is the epigenetic control of oxidative and nitrosative stress responses, which constitute core components of innate immune killing. In *Pseudomonas aeruginosa*, the orphan MTase M.PaeTBCFII contributes to resistance to reactive nitrogen species that are produced by macrophages, with deletion mutants exhibiting reduced intracellular survival (Han et al. 2022). Similarly, in *Haemophilus influenzae*, immune pressure imposed by neutrophil oxidative bursts rapidly enriches *modA2*-OFF subpopulations, demonstrating how phase-variable methylation generates immune-adapted phenotypes without genetic change (Brockman et al. 2017). In *Salmonella typhimurium*, Dam deficiency disrupts *oxyR* and *rpoS*-dependent stress regulons, resulting in impaired survival under oxidative challenge and reduced persistence in murine lymphoid tissues *in vivo* (Balbontín et al. 2006, Zhang et al. 2023). Phase-variable ModD systems in *Neisseria meningitidis* further link methylation to immune resistance, with ModD-ON variants exhibiting increased catalase activity and enhanced survival following oxidative bursts (Seib et al. 2011). Type I R–M system SpyMEW123I *Streptococcus pyogenes* modulates infection and host inflammatory response, ΔSpyMEW123I mutant exhibited larger lesions and elevated levels of interleukins from skin biopsies in murine infection models (Nye TM et al. 2019).

Methylation can also affect intracellular trafficking and phagosome maturation, thereby promoting long-term persistence. An example is provided by the MTase Rv1509 in pathogenic mycobacteria. Expression of Rv1509 alters global transcription, including ESX-1 secretion system genes, inhibits phagolysosomal fusion, maintains bacteria within Rab5-positive early endosomal compartments, and suppresses nitric oxide production by macrophages (Manjunath et al. 2024).

Immune evasion is further mediated through methylation-dependent remodeling of cell surface structures, altering host recognition, and inflammatory outcomes. In *Yersinia enterocolitica*, Dam overproduction modifies lipid A core structures, increasing exposure of the Inv adhesin and enhancing epithelial invasion under low-temperature conditions while simultaneously hindering innate immune recognition (Fälker et al. 2005, Fälker et al. 2007). In *N. gonorrhoeae*, ModA13 switching also alters biofilm architecture, with ModA13-OFF variants forming denser biofilms that provide enhanced shielding from immune effectors and support tissue persistence (Srikhanta et al. 2009).

In many organisms, virulence modulation arises indirectly through global metabolic changes. In *Vibrio cholerae*, VchM contributes to intestinal colonization and drug susceptibility through broader physiological methylation-driven shifts (see Section 4.4) (Chao et al. 2015, Carvalho et al. 2021). Similarly, in the entomopathogen *Photorhabdus luminescens*, Dam overexpression slows host killing kinetics during 2 insect infestation models, which has been associated with altered pathogenicity of nemato-bacterial complex (Payelleville et al. 2019). These examples demonstrate that methylation can modulate not only virulence severity but also the rate of pathogenesis.

Phase-variable Type I R–M systems further expand virulence potential by generating allele-specific pathogenic programs within clonal populations. In *Listeria monocytogenes*, locked *hsdS* alleles produce distinct methylation landscapes and markedly different disease outcomes in neonatal rat CNS infection models, with individual alleles conferring advantages in specific anatomical niches. To expand on this, allele C is associated with the most severe neuropathology, including pronounced hippocampal apoptosis, whereas allele D yields high cerebellar burdens and substantial weight loss; allele A exhibits the least virulence (Zbinden et al. 2020). Mixed-infection experiments reveal competitive advantages for particular epigenetic states *in vivo*, underscoring the role of methylation diversity in within-host adaptation (Zbinden et al. 2020). Similarly, in *Streptococcus suis*, the SsuCC20p phase-variable system switches among *hsdS* alleles that modulate nutrient uptake and biosynthetic pathways, yielding epigenetically encoded virulence states with distinct replication fitness and mortality outcomes in zebrafish larvae (Roodsant et al. 2024).

These systems demonstrate that DNA methylation does not usually operate as a singular virulence determinant, but rather as a regulatory framework that coordinates surface architecture, secretion systems, metabolic state, stress tolerance, and immune engagement. By enabling reversible transitions between colonizing, invasive, and persistent states without genetic change, methylation provides pathogens with a powerful mechanism for adaptive plasticity during infection.

### Stress response and environmental resilience

Whereas Section 4.1 emphasized stress responses in the context of host immunity, here we focus on methylation-mediated stress adaptation as a general ecological and environmental survival strategy. These states influence oxidative, osmotic, envelope, desiccation, and chemical stresses encountered in environmental niches. Through remodelling of stress response networks, methylation can mediate survival of bacterial cells under fluctuating or hostile environments. Representative examples of methylation-dependent stress response and resilience across prokaryotes are summarized in Table 2.

**Table 2:** Stress response and environmental resilience linked to DNA methyltransferases (Section 4.2).

MTase category	Species	MTase state/manipulation	Stress-associated functional outcome	Reference
Orphan MTase (Dam)	*Salmonella typhimurium*	Deletion mutant	Impaired oxidative stress survival	Zhang et al. 2023
Phase-variable MTase (ModD)	*Neisseria meningitidis*	ON/OFF variants	State-dependent resistance to oxidative stress	Seib et al. 2011
Orphan MTases (XvDMT1/2)	*Xanthomonas euvesicatoria*	Overexpression mutant	Altered tolerance to ethanol/sorbitol and polymyxin B	Park et al. 2021
Orphan MTase (EadM)	*Xanthomonas axonopodis*	Overexpression mutant	Enhanced desiccation resistance and sorbitol and ciprofloxacin susceptibility	Park et al. 2019
Phase-variable MTase (ModS2)	*Streptococcus suis*	ON/OFF switching	Differential tolerance to β-lactam antibiotics	Tram et al. 2021
Phase-variable MTase (ModA13)	*Neisseria gonorrhoeae*	ON/OFF variants	Differential resistance to detergent stress	Srikhanta et al. 2009
Cytosine MTase (LomA)	*Leptospira interrogans*	Deletion mutant	Impaired growth-phase transition and envelope resilience	Gaultney et al. 2020
Restriction–modification system (SuaI)	*Sulfolobus islandicus*	Deletion mutant	Reduced genome stability under thermal stress, increased transformation efficiency	Suzuki and Kurosawa 2016
Virus-encoded Dam-like MTase	Halophilic archaea	Heterologous expression	Modulation of osmotic stress tolerance	Baranyi et al. 2000

A recurring feature across taxa is methylation-dependent modulation of oxidative stress responses, which might be critical for the growth under various environmental conditions. As mentioned previously *S. typhimurium*, Dam methylation through OxyR/RpoS supports survival during peroxide exposure, with *dam* mutants exhibiting pronounced survival defects across multiple H₂O₂ concentrations. Notably, methylation levels at GATC sites fluctuate within minutes of oxidative challenge, suggesting that Dam participates in rapid epigenetically mediated adaptation (Zhang et al. 2023). Comparable regulation is observed in *N. meningitidis*, where ModD-ON variants exhibit enhanced resistance to H₂O₂, traits that likely support survival during environmental transmission and colonization under unfavourable conditions (Seib et al. 2011).

Beyond redox stress, DNA methylation can contribute to stress tolerance of other sources. In plant pathogens such as *Xanthomonas euvesicatoria*, overexpression of the MTases XvDMT1 or XvDMT2 generates divergent stress-tolerance profiles. XvDMT1 enhances survival under ethanol and high-sorbitol osmotic stress, whereas XvDMT2 substantially reduces ethanol and sorbitol tolerance but enhances polymyxin B tolerance (Park et al. 2021). Overexpression of *eadM* in *Xanthomonas axonopodis* reduces stress resilience to ciprofloxacin and D-sorbitol but increases siderophore production under iron limitation (Park et al. 2019).

Methylation-dependent stress adaptation also extends to antibiotic tolerance, particularly through indirect effects on protein folding, envelope maintenance, and cellular homeostasis. In *S. suis*, ModS2-OFF variants display enhanced tolerance to β-lactam antibiotics, consistent with methylation-dependent tuning of cell wall biosynthesis enzymes and stress regulators (Tram et al. 2021). Similarly, in *N. gonorrhoeae*, ModA13 phase variation affects resistance to detergents such as Triton X-100 via regulation of LPS modification pathways (Srikhanta et al. 2009). In the swine pathogen *Actinobacillus pleuropneumoniae*, the ModP2-ON phasevarion also exhibits increased resistance to several antibiotics commonly used to treat porcine infections, including ampicillin, penicillin, florfenicol, tulathromycin, tilmicosin, and tiamulin (Nahar et al. 2023).

DNA methylation also contributes to resilience under desiccation, temperature extremes, and other harsh environmental conditions, particularly in soil, aquatic, and plant-associated microbes. In *X. axonopodis*, EadM overexpression enhances resistance to desiccation despite compromising other stress responses, illustrating the specificity with which methylation can allocate resources toward particular survival strategies (Park et al. 2019). Similarly, in *Leptospira interrogans*, loss of the cytosine MTase LomA delays lag-phase exit, reduces motility through semi-solid media, and increases susceptibility to polymyxin B (Gaultney et al. 2020). By contrast, in *Shewanella oneidensis* MR-1, comparative methylome analyses under aerobic and anaerobic growth conditions reveal subtle variation in DNA methylation, consistent with a limited role as a primary transcriptional regulator in this species. Instead, enrichment of methylated motifs near the origin of replication points to a possible role in replication-associated genome organization rather than stress-responsive gene control (Bendall et al. 2013).

In extremophilic archaea, methylation-mediated stress adaptation takes on additional dimensions. In the hyperthermophile *Sulfolobus islandicus*, the SuaI R–M system methylates cytosine residues and appears to contribute to genome stability at high temperatures, where spontaneous cytosine deamination is frequent. Notably, N4-cytosine methylation is thought to be favoured in many thermophilic archaea because cytosine methylation at the C5 position promotes spontaneous deamination to thymine, whereas methylation at the N4 position reduces the rate of cytosine deamination, thereby enhancing genome stability at elevated temperatures (Ehrlich et al. 1986). Loss of methylation increases mutation rates and reduces viability, representing a rare case in which DNA methylation directly supports genome stability at high temperatures (Grogan 2003, Suzuki and Kurosawa 2016). In halophilic archaea, virus-encoded Dam-like MTases such as M.ϕCh1-I modify adenine residues and can complement *E. coli* Dam function under low-salt conditions, revealing how horizontally acquired MTases may enhance osmotic resilience (Baranyi et al. 2000).

Finally, epigenetic heterogeneity generated by phase-variable MTases provides a powerful mechanism for stress adaptation. Mod systems in *Neisseria* spp., ModS in *S. suis*, and ModA2 in *H. influenzae* generate ON/OFF subpopulations with distinct stress tolerance profiles, enabling rapid shifts in population structure (Srikhanta et al. 2009, Jen et al. 2014, Brockman et al. 2017, Tram et al. 2021). Under oxidative stress, for example, *modA2*-OFF *H. influenzae* cells rapidly dominate mixed cultures, illustrating how methylation-driven phenotypic diversification maximizes survival without requiring genetic mutation (Brockman et al. 2017).

Within environmental contexts, methylation coordinates multiple stress-response pathways through both stable orphan MTases and dynamic phase-variable systems, enabling survival under fluctuating and extreme conditions.

### Horizontal gene transfer and evolutionary adaptation

DNA methylation is a major architect of genome plasticity in bacteria and archaea. By shaping recombination rates, modulating DNA uptake, controlling the integration of mobile genetic elements, and influencing mutation trajectories, methyltransferases (MTases) may act as gatekeepers of evolutionary potential. Representative examples of methylation-dependent alteration of horizontal gene transfer (DNA uptake) and evolution across prokaryotes are summarized in Table 3.

**Table 3:** Roles of DNA methyltransferases in horizontal gene transfer and evolutionary adaptation (Section 4.3).

MTase category	Species	MTase state or manipulation	Evolutionary/HGT-associated functional outcome	Reference
Phase-variable Type I R–M (SsuCC20p)	*Streptococcus suis*	ON/OFF variants	Lineage-specific filtering of horizontally acquired DNA	Willemse et al. 2016, Atack et al. 2018
Cytosine MTase	*Synechocystis* sp. PCC 6803	Deletion mutant	Altered conjugation efficiency without loss of CRISPR immunity	Scholz et al. 2019
Orphan MTase (Dam)	*Yersinia enterocolitica*	Overexpression mutant	Increased spontaneous mutation rate	Fälker et al. 2005
Phase-variable MTases	*Haemophilus influenzae*	ON/OFF variants	Indirect promotion of surface antigen diversification and population heterogeneity in DNA acquisition	Brockman et al. 2017
Phase-variable MTase (ModA13)	*Neisseria gonorrhoeae*	ON/OFF variants	Differential permissiveness to phage adsorption and plasmid compatibility	Srikhanta et al. 2009
Phage-encoded MTase (PamA)	*Staphylococcus aureus*	Prophage-encoded expression	Modulation of host surface traits influencing phage fitness	Ulrich et al. 2024
Orphan MTases (EadM, XvDMT1/2)	*Xanthomonas* spp.	Overexpression mutant	Altered plasmid stability and phage susceptibility	Park et al. 2019, 2021
Orphan MTase (MamA)	*Mycobacterium tuberculosis*	Deletion mutant	Reduced persistence under hypoxia-linked selective pressure	Shell et al. 2013
Orphan MTase (Rv1509)	Pathogenic mycobacteria	Expressed in *M. smegmatis (Transformation)*	Transcriptional and morphological states linked to long-term adaptation	Manjunath et al. 2024

One of the most direct ways in which methylation influences genome evolution is through the interaction of R–M systems with foreign DNA. Type I and Type III R–M systems function not only as barriers to phage and plasmid entry but also as selective filters that determine which genetic elements can be assimilated. In *S. suis*, the phase-variable Type I system SsuCC20p has played a central role in shaping the genomic trajectory of the zoonotic CC20 lineage (Atack et al. 2018). Acquired through horizontal transfer, this system establishes a lineage-specific methylation landscape that filters incoming mobile elements, including capsule loci and regulatory genes, thereby stabilizing accessory regions that define clonal identity and interspecies transmission potential. More broadly, distinct methyltransferase repertoires segregate with clonal complexes across the genus, indicating that methylation systems might contribute to lineage diversification by modulating DNA acquisition and expression of newly integrated genes (Willemse et al. 2016, Atack et al. 2018, Roodsant et al. 2024). Phages represent one of the most prominent sources of foreign DNA encountered by R–M systems. Using a multistage infection model with *Helicobacter pylori* and phage KHP30 (genus *Schmidvirus*), a study showed that phages produced high titers when infecting their most recent host strain but significantly lower titers against other strains. This strain-specific infectivity suggests that phages can evade host R–M barriers by acquiring host-derived methylation patterns during replication (Takahashi et al. 2025).

DNA methylation can modulate the efficiency and directionality of horizontal gene transfer through effects on transformation and conjugation. In the cyanobacterium *Synechocystis* sp. PCC 6803, disruption of cytosine methylation alters conjugation efficiency without impairing CRISPR-Cas immunity (Scholz et al. 2019). Dense methylation of CRISPR repeats and relative hypomethylation of *cas* loci appear to influence the structural organization or processing of these regions during DNA uptake, representing a rare example of methylation affecting gene transfer through non-transcriptional mechanisms (Scholz et al. 2019). Such effects shape not only the frequency of DNA acquisition but also the timing of population-level genetic exchange.

Beyond DNA uptake, methylation influences genome stability and mutation rates. Dysregulation of adenine methylation can increase mutability, the overproduction of Dam in *Y. enterocolitica* elevates spontaneous mutation rates, highlighting the dual nature of methylation as either genome-protective or mutagenic depending on concentration and context (Fälker et al. 2005). In species with extensive phase-variation, such as *Neisseria* and *Haemophilus*, ON/OFF switching of *mod* genes alters the exposure of simple sequence repeats, indirectly promoting genetic instability and diversification of surface structures critical for niche adaptation (Bayliss et al. 2006, Tan et al. 2016).

Methylation further shapes genome plasticity by influencing recombination and mobile element activity, including phage integration, capsule switching, and plasmid maintenance. In *N. gonorrhoeae*, ModA13-mediated modulation of LPS modification genes and outer membrane structures generates phenotypes that differentially permit phage adsorption and plasmid compatibility (Srikhanta et al. 2009). In *Staphylococcus aureus*, the phage-encoded MTase PamA modulates expression of host surface proteins such as FnBPA, suggesting a feedback loop in which phage-derived MTases influences host phenotype (Ulrich et al. 2025). In plant pathogen *Xanthomonas* spp., overexpression of MTases such as EadM or XvDMT1/2 strongly influences plasmid stability and phage susceptibility in natural environments (Park et al. 2019, 2021). In *Mycoplasma agalactiae*, methylome remodeling driven by multiple DNA methylation systems reshapes the epigenomic landscape of mycoplasma and subsequently host adaptation, providing an example of epigenetically driven physiological plasticity (Dordet-Frisoni et al. 2022).

A further dimension of methylation-driven evolution arises from methylome heterogeneity, particularly through phase-variable MTases that generate subpopulations with distinct regulatory and genomic states. These epigenetic clusters differ not only in gene expression but also in permissiveness to recombination and horizontal gene transfer. In *S. suis, N. meningitidis*, and *H. influenzae*, alternative ON and OFF states produce populations that vary in capsule expression, antibiotic tolerance, and stress resilience traits that directly influence clonal success under specific ecological or clinical conditions (Srikhanta et al. 2009, Brockman et al. 2017, Tram et al. 2021). Because these epigenetic states switch at high frequency, phase-variable MTases may act as evolutionary accelerators, enabling rapid exploration of phenotypic and genomic space without requiring permanent genetic change.

Finally, methyltransferases contribute to long-term evolutionary adaptation by coordinating large-scale transcriptional remodeling during environmental transitions and selective pressures. In *Mycobacterium tuberculosis*, the MamA MTase promotes persistence under hypoxic conditions characteristic of granulomatous lesions, while Rv1509 reshapes cell morphology, ribosomal content, and phagosome maturation, generating phenotypes associated with intracellular persistence and slow growth (Shell et al. 2013, Manjunath et al. 2024). These methylation-driven states enhance survival through ecological bottlenecks that ultimately shape evolutionary success.

Across bacteria and archaea, DNA methylation thus serves as a multilayered determinant of genome plasticity: filtering DNA acquisition, shaping mutation landscapes, stabilizing genomes under stress, generating reversible heterogeneity, and altering the fitness consequences of horizontal gene flow. Through these mechanisms, methyltransferases exert profound influence over both short-term adaptation and long-term evolutionary trajectories, functioning as active drivers of microbial diversification.

### Metabolic regulation and growth dynamics

DNA methyltransferases exert influence over bacterial metabolic programs and growth dynamics by modulating networks that govern nutrient acquisition, energy generation, anabolic capacity, and cellular architecture. Representative examples of methylation-dependent metabolic modulation and altered growth across prokaryotes are summarized in Table 4.

**Table 4:** Metabolic regulation and growth dynamics controlled by DNA methyltransferases (Section 4.4).

MTase category	Species	MTase state or manipulation	Metabolic/growth-associated functional outcome	Reference
Cytosine MTase (VchM)	*Vibrio cholerae*	Deletion mutant	Global metabolic changes and impaired growth	Chao et al. 2015
Cytosine MTase (VchM)	*Vibrio cholerae*	Deletion mutant	Altered envelope physiology and increased aminoglycoside tolerance	Carvalho et al. 2021
Type I R–M system (HsdMSR)	*Pseudomonas syringae*	Deletion mutant	Broad alteration of central metabolism and redox balance	Huang et al. 2025
Orphan MTase (EadM)	*Xanthomonas axonopodis*	Overexpression mutant	Compromised growth with widespread metabolic and proteomic fluctuations	Park et al. 2019
Orphan MTases (XvDMT1/2)	*Xanthomonas euvesicatoria*	Overexpression mutant	Carbon source-dependent growth defects and altered carbohydrate flux	Park et al. 2021
Orphan MTase (M.PaeTBCFII)	*Pseudomonas aeruginosa*	Deletion mutant	Impaired denitrification-linked energy metabolism	Han et al. 2022
Orphan MTase (DamA)	*Streptococcus mutans*	Deletion mutant	Altered carbohydrate uptake and extracellular polysaccharide synthesis	Banas et al. 2011
Orphan MTase (MamA)	*Mycobacterium tuberculosis*	Deletion mutant	Reduced survival under hypoxia-associated metabolic stress	Shell et al. 2013
Orphan MTase (Rv1509)	Mycobacterium spp.	Expressed in *M. smegmatis (Transformation)*	Global shifts in metabolism, growth rate, and cell morphology	Manjunath et al. 2024
Cytosine MTase (LomA)	*Leptospira interrogans*	Deletion mutant	Disrupted growth-phase transitions and membrane-associated metabolism	Gaultney et al. 2020

An illustration of the link between methylation and metabolic fitness is provided by *Vibrio cholerae*. The cytosine methyltransferase VchM is required for efficient proliferation under both aerobic and anaerobic conditions, and *ΔvchM* mutants display distinctive transcriptional profiles in amino-acid biosynthesis, carbohydrate metabolism, and respiratory pathways. Disruption of LPS and envelope-associated gene expression further compromises viability *in vivo* (Chao et al. 2015). In parallel, VchM deficiency enhances tolerance to aminoglycosides through upregulation of chaperone systems such as GroEL/GroES, highlighting how methylation-mediated metabolic tuning can simultaneously influence growth capacity and antibiotic resilience (Carvalho et al. 2021). Similar links between methylation patterns and metabolic adaptation have also been observed in environmental microbial communities. Metagenomic analyses of stratified sea-ice microbial communities have revealed distinct methylation signatures associated with different ice layers. In *Pelagibacter* metagenome-assembled genomes, orphan methyltransferases and a prophage-associated Type II methyltransferase targeting GGATG motifs suggest potential regulatory roles, while differential methylation at GANTC motifs by orphan MTases has been associated with genes involved in metabolic processes and growth, consistent with possible epigenetic responses to the fluctuating environmental conditions of upper sea-ice layers (Kanaan and Deming 2025).

Metabolic reprogramming driven by methylation is also prominent in plant-associated bacteria. In *P. syringae*, the HsdMSR system regulates hundreds of genes involved in redox balance, central metabolism, and environmental signalling; traits essential for survival on plant surfaces and within host tissues (Huang et al. 2025). Comparable effects are observed in *Xanthomonas* spp., where overexpression of methyltransferases such as EadM in *X. axonopodis* pv. *glycines* or XvDMT1/2 in *X. euvesicatoria* imposes widespread physiological changes. EadM overexpression compromises growth both in plants and *in vitro*, accompanied by large-scale proteomic shifts affecting envelope biosynthesis, siderophore production, sugar utilization, and oxidative metabolism (Park et al. 2019, 2021). In *X. euvesicatoria*, XvDMT1/2 alters carbon source-dependent growth, leading to impaired proliferation with sucrose or fructose, reduced exopolysaccharide production, and changes in the abundance of the phosphocarrier protein HPr (Park et al. 2021). Together, these findings demonstrate that DNA methylation can alter central carbohydrate flux and metabolic routing, rather than simply suppressing growth.

Nitrogen metabolism was also found to be modulated by DNA methylation. In *P. aeruginosa*, the orphan adenine MTase M.PaeTBCFII regulates genes involved in denitrification, including *nosR* and *norC*. Deletion mutants show reduced expression of these pathways, while complemented strains display expression levels exceeding those of the wild type, indicating methylation dependence of nitrogen redox regulation (Han et al. 2022). Because denitrification supports energy generation in oxygen-limited environments, these effects directly impact growth potential and survival in niches such as biofilms and intracellular compartments.

In Gram-positive bacteria, methylation also governs carbohydrate metabolism and biosynthetic output. In *Streptococcus mutans*, disruption of *damA* triggers extensive alteration of sugar transport and catabolic pathways, including strong upregulation of the cellobiose phosphotransferase system, multiple bacteriocin loci, and glucosyltransferases (*gtfB* and *gtfC*). These changes alter extracellular polysaccharide production, colony morphology, and mutacin activity, highlighting metabolic tuning as a central methylation-controlled determinant of ecological fitness within the oral cavity (Banas et al. 2011).

Several intracellular and slow-growing pathogens exhibit particularly strong coupling between methylation and metabolic adaptation. In *M. tuberculosis*, deletion of the essential MTase MamA keeps *in vitro* growth and oxidative or nitrosative stress responses largely intact but severely compromises survival under hypoxic conditions. Transcriptomic analyses reveal repression of stress-responsive and metabolic regulators, indicating that MamA is required to remodel respiratory and metabolic pathways for persistence in oxygen-depleted, nutrient-limited environments (Shell et al. 2013). A related methyltransferase, Rv1509, induces global shifts in tRNA abundance, metabolic gene expression, cell morphology, and envelope architecture when expressed in *Mycobacterium smegmatis*, resulting in slowed growth and enhanced intracellular survival (Manjunath et al. 2024).

Comparable methylation-linked metabolic effects are observed in environmental and soil-associated bacteria. In *Leptospira interrogans*, loss of the cytosine MTase LomA disrupts growth-phase transitions, reduces motility, and alters membrane integrity, reflecting methylation-mediated control over energy utilization and envelope physiology (Gaultney et al. 2020).

Across these diverse taxa, several unifying themes emerge. DNA methyltransferases mainly modulate metabolic networks not through isolated promoter-level effects but via broad transcriptional and proteomic reconfiguration. By tuning energy generation, nutrient uptake, and structural integrity, methylation aligns cellular physiology with environmental and ecological constraints. These findings indicate that DNA methylation can be pivotal as a global physiological integrator that regulates metabolism, growth dynamics, and ecological fitness.

### Cellular adhesion, biofilm formation, and development

Biofilm formation represents one of the most complex emergent phenotypes influenced by DNA methylation, integrating adhesion, extracellular matrix production, surface sensing, and multicellular organization. Because biofilms function as protective niches that promote environmental persistence, antimicrobial tolerance, and chronic infection (Boudet et al. 2021), methylation-dependent control of biofilm traits carries broad ecological and clinical significance Across bacteria and archaea, MTases influence biofilm formation and development by coordinating various cellular processes including motility, cell adhesion, quorum sensing, as well as extracellular matrix production. Representative examples of methylation-dependent modification in biofilm formation or structure across prokaryotes are summarized in Table 5.

**Table 5:** DNA methyltransferase-dependent regulation of cellular adhesion, biofilm formation, and development (Section 4.5).

MTase category	Species	MTase state or manipulation	Biofilm-associated functional outcome	Reference
Orphan MTase (M.NgoAX)	*Neisseria gonorrhoeae*	Deletion mutant	Altered early surface attachment and microcolony architecture	Kwiatek et al. 2015
Phase-variable Type III MTase (ModA13)	*Neisseria gonorrhoeae*	ON/OFF variants	State-dependent biofilm architecture (dense vs sparse)	Srikhanta et al. 2009
Phase-variable Type I R–M systems (Spn556II, SpnD39III)	*Streptococcus pneumoniae*	ON/OFF variants	Indirect modulation of biofilm assembly via surface and metabolic traits	Agnew et al. 2023
Phase-variable Type III MTase (ModM)	*Moraxella catarrhalis*	ON/OFF variants	Remodeling of protein networks supporting mature biofilm formation	Blakeway et al. 2014
Phage-encoded MTase (PamA)	*Staphylococcus aureus*	Prophage-encoded expression	Increased fibronectin-mediated adhesion and biofilm biomass *in vivo*	Ulrich et al. 2024
Orphan MTase (EadM)	*Xanthomonas axonopodis* pv. *glycines*	Overexpression mutant	Impaired biofilm formation linked to altered envelope stability	Park et al. 2019
Orphan MTase (XvDMT2)	*Xanthomonas euvesicatoria*	Overexpression mutant	Reduced biofilm formation under carbohydrate-rich conditions	Park et al. 2021
Orphan MTase (Dam)	*Photorhabdus luminescens*	Overexpression mutant	Increased biofilm biomass with reduced motility	Payelleville et al. 2017
Orphan MTase (*damA*)	*Streptococcus mutans*	Deletion mutant	Enhanced extracellular polysaccharide production and altered biofilm architecture	Banas et al. 2011)
Undefined MTases	*Sulfolobus* spp.	Methylation-dependent regulation	Modulation of surface glycosylation and community-associated traits	Reva et al. 2024

In *N. gonorrhoeae*, where both phase-variable and constitutively expressed MTases remodel community architecture, the loss of the non-phase-variable MTase M.NgoAX reduces early surface attachment, while it promotes the formation of compact, clumped microcolonies, indicative of denser biofilm structures with reduced membrane blebbing. This is associated with the DNA methylation-dependent downregulation of pilus-associated genes and adhesins (Kwiatek et al. 2015). Complementary effects are observed in the ModA13 phasevarion, where ON and OFF states generate markedly distinct biofilm architectures; *modA13*-OFF populations form thick, cohesive biofilms, whereas *modA13*-ON variants produce sparse, fragile structures (Srikhanta et al. 2009), in association to a modulation of the expression of genes involved in surface proteins, oxidative stress defences, and efflux pathways.

In other bacteria methylation indirectly shapes biofilm behaviour by modifying surface architecture and metabolic context. In *S. pneumoniae*, Type I phase-variable systems such as Spn556II and SpnD39III regulate capsule production, colony opacity, and carbon source utilization. While these effects primarily influence adhesion and colonization (Section 4.1), they secondarily impact biofilm assembly by altering surface charge, matrix interactions, and persistence within nasopharyngeal communities (Agnew et al. 2023, Li et al. 2025 ). Similarly, the ModM phasevarion of *Moraxella catarrhalis*, which differs in prevalence between nasopharyngeal and middle-ear isolates, modulates protein networks involved in envelope maintenance and nutrient acquisition key determinants of mature biofilm structure during otitis media (Blakeway et al. 2014).

In *S. aureus*, the phage-encoded Dam-like MTase PamA upregulates *fnbA* more than fifteen-fold, increasing fibronectin-binding capacity and producing a six-fold increase in biofilm biomass during *in vivo* abscess formation. (Ulrich et al. 2025). Type II-G R–M system “*cj0031*” affects biofilm biomass, *cj0031-OFF* and deletion mutant experienced a four to seven-fold reduction in biomass as compared to *cj0031-ON* in *C. jejuni (*Anjum et al. 2016). In the swine pathogen *Actinobacillus pleuropneumoniae*, a Type III cytosine-specific R–M system also affects growth and biofilm development, with the ModP1-ON variant displaying increased growth rates and greater biofilm biomass relative to the isogenic ModP1-OFF strain (Nahar et al. 2023).

In *Burkholderia cenocepacia*, a Type III R–M system “BCAL3494” was found to drive biofilm structural changes, deletion mutants of BCAL3494 (ΔBCAL3494) exhibited biofilms of a more clustered morphology compared to the wildtype under microscopic analysis. ΔBCAL3494 mutants were also prone to forming cellular aggregates with higher pellicle formation when in planktonic growth compared to the wildtype as tested by flow cytometry and crystal violet staining of the pellicle (Vandenbussche et al. 2020). In *S. mutans*, disruption of *damA* increases expression of glucosyltransferases (*gtfB* and *gtfC*) and glucan-binding protein GbpC, driving elevated extracellular polysaccharide production and altering the three-dimensional architecture of dental plaque biofilms (Banas et al. 2011).

Plant-associated pathogens further illustrate how methylation regulates biofilm traits through control of envelope integrity and carbohydrate flux. In *X. axonopodis* pv. *glycines*, overexpression of the MTase EadM alters envelope stability and exopolysaccharide production, compromising the bacterium’s ability to assemble robust surface-associated communities despite enhanced siderophore secretion (Park et al. 2019). In *X. euvesicatoria*, overexpression of XvDMT2 reduces biofilm formation and exopolysaccharide production indicating a possible link to matrix biosynthesis and surface attachment (Park et al. 2021). Methylation-dependent biofilm regulation is also evident in animal and insect-associated bacteria. In *P. luminescens*, Dam overexpression enhances biofilm biomass while suppressing motility, revealing an inverse relationship between dispersal and sessility that is encoded within methylation-responsive regulatory networks (Payelleville et al. 2017). These changes might promote community cohesion while reshaping competitive interactions within multispecies biofilms.

In archaea, evidence for methylation-mediated biofilm regulation is emerging. In *Rv15olobus* spp., methylation alters the regulation of genes involved in surface glycosylation, S-layer stability, and environmental tolerance, traits central to archaeal community formation in acidic, high-temperature habitats (Reva et al. 2024). While direct functional validation of biofilm phenotypes is still sparse, these observations suggest that epigenetic regulation of surface-associated behaviours may extend beyond bacteria.

A consistent theme emerges: DNA methylation governs biofilm-related phenotypes not through single effector pathways but via coordinated modulation of surface properties, metabolic context, stress tolerance, and motility-adhesion trade-offs. By tuning these interconnected modules, methyltransferases determine whether cells disperse, aggregate, or commit to sessile growth.

## Conclusions and future directions

DNA MTases have emerged as key regulators of microbial physiology, influencing virulence, stress tolerance, genome plasticity, host interaction, and ecological fitness across bacteria and archaea. The central challenge now is to translate expanding methylome catalogues into mechanistic understanding and ultimately predictive models of MTase-driven phenotypes.

Based on compiled evidence, DNA methylation functions as a multifaceted epigenetic system that links gene regulation, genome organization, and physiological state to microbial fitness across diverse ecological and host-associated environments. Rather than serving as a universal transcriptional regulator, methyltransferases act through species and context-dependent mechanisms that range from local, site-specific effects to genome-wide remodelling of methylation patterns with broad regulatory consequences. Through these processes, DNA methylation influences virulence, stress resilience, metabolic adaptation, biofilm development, horizontal gene transfer, and evolutionary trajectories, frequently by coordinating trade-offs between growth, persistence, and adaptability. A recurring feature across systems is the generation of reversible phenotypic heterogeneity most prominently via phase-variable methyltransferases, which enable rapid population-level responses to shifts in environmental conditions without requiring permanent genetic change.

A critical need is tighter integration of methylome mapping with functional validation. Over the last decade, SMRT and nanopore sequencing have greatly expanded information on DNA methylation levels and motifs in prokaryotic genomes; however, only a subset of these studies connects methylation patterns to gene expression changes and measurable phenotypes. This gap is evident in systems where regulatory conclusions are inferred primarily from motif distributions or correlative transcriptomic data. Future work should more routinely pair methylome profiling with (i) MTase perturbation (deletion, enzyme inactive mutants, controlled expression), (ii) allele locking for phase-variable systems, and (iii) quantitative phenotyping across relevant host and environmental conditions. Longitudinal observation through evolutionary studies that can track methylation states during infection, biofilm development, and stress transitions will be especially valuable for distinguishing whether methylation changes are causal, compensatory, or incidental.

A second frontier lies in understanding MTases as drivers of genome plasticity and evolutionary change. Phase-variable MTases generate epigenetically distinct subpopulations that can be differentially favoured across niches, while R–M systems shape the permeability of genomes to horizontal gene transfer. Future studies combining experimental evolution, comparative methylomics and population-genetic modelling should clarify when MTase switching primarily functions as short-term population-level phenotypic diversification versus when it produces durable lineage-level consequences (e.g. by controlling mobile element acquisition, capsule switching, or phage susceptibility).

Furthermore, for several organisms and MTase systems, the evidence base remains narrow, sometimes resting on a small number of studies from single laboratories. This creates both an opportunity and a risk. Indeed, these findings may represent broadly conserved principles, but they also require independent replication and extension across strains, conditions and infection models. Community efforts to standardize methylome reporting, deposit raw modification calls, and adopt shared phenotyping benchmarks would help consolidate robust, comparable evidence.

The functional centrality of MTases also raises the prospect of using these systems as antimicrobial targets. In multiple pathogens, disruption or dysregulation of particular MTases can reduce virulence, weaken stress defences or increase immune susceptibility, suggesting that MTase inhibition might destabilize transcriptional homeostasis and sensitize bacteria to host clearance or antibiotic treatment. Realizing this potential will require careful prioritization of targets (to avoid rapid bypass mechanisms), deeper understanding of off-target effects on commensals, and improved structural insight to support selective inhibitor design.

Last, beyond their natural roles, MTases are gaining attention as programmable molecular tools in synthetic biology and microbial engineering. Emerging demonstrations that MTase-driven methylation can encode reversible regulatory states highlight a design space for orthogonal epigenetic control, including stable memory modules, circuit insulation and controllable barriers to gene transfer including tools such as biosensors (Graf et al. 2023). Expanding this toolkit will depend on defining specificity determinants, building libraries of target recognition domains, and developing reliable approaches for tuning MTase activity without global fitness costs.
